# Comprehensive reconstruction of the musculoskeletal anatomy in the shoulder using a hybrid 3D ultrasound mosaicking workflow: A pilot study

**DOI:** 10.1371/journal.pone.0347231

**Published:** 2026-06-09

**Authors:** Ahmed Sewify, Marian Steffens, Naomi Perrier, Maria Antico, Maxence Lavaill, Christopher Edwards, Peter Pivonka, Davide Fontanarosa

**Affiliations:** 1 School of Clinical Sciences, Queensland University of Technology, Gardens Point Campus, Brisbane, Queensland, Australia; 2 Centre for Biomedical Technologies, Queensland University of Technology, Brisbane, Queensland, Australia; 3 Australian e-Health Research Centre, The Commonwealth Scientific and Industrial Research Organisation (CSIRO), Herston, Queensland, Australia; 4 School of Mechanical Medical and Process Engineering, Faculty of Engineering, Queensland University of Technology, Gardens Point Campus, Brisbane, Queensland, Australia; Carol Davila University of Medicine and Pharmacy: Universitatea de Medicina si Farmacie Carol Davila din Bucuresti, ROMANIA

## Abstract

**Background:**

Three-dimensional (3D) ultrasound (US) imaging of complex joints such as the shoulder is limited by a small field of view and shallow imaging depth. As a result, capturing the entire shoulder anatomy in a single scan is not feasible. This study evaluates a hybrid 3D US mosaicking workflow that combines probe pose tracking, image-based alignment, and magnetic resonance imaging (MRI) guidance to reconstruct the full shoulder musculoskeletal anatomy.

**Methods:**

Five healthy volunteers underwent shoulder imaging using a robot-assisted 3D US probe. Each shoulder was divided into five scan regions, yielding approximately 45 partially overlapping US volumes per trial. Volumes were first positioned using probe pose information and then refined using a hybrid alignment strategy that combined semi-automatic US-US registration with expert manual adjustments guided by subject-specific MRI bone models. Reconstruction performance was assessed by measuring alignment consistency of the humerus across overlapping volumes using Dice similarity coefficients and multimodal overlap metrics.

**Results:**

The hybrid workflow improved reconstruction accuracy by approximately 17% compared to pose estimation alone. The refined reconstructions showed clearer anatomical continuity and fewer alignment artefacts. Reconstruction accuracy was influenced by the amount of overlap between scans and by differences in shoulder position between US and MRI acquisitions. Weighted mean compounding performed best for regional reconstructions with sufficient overlap, while maximum compounding was more effective for whole-shoulder mosaics.

**Conclusion:**

This study demonstrates the feasibility of reconstructing the full shoulder anatomy using a hybrid 3D US mosaicking approach. The proposed workflow improves alignment accuracy and anatomical visualisation compared with pose estimation alone and provides a practical reference framework for future large-scale and automated, real-time musculoskeletal US reconstruction.

## 1. Introduction

Ultrasound imaging (US) is currently the only non-invasive medical modality capable of volumetric (3D) real-time imaging, facilitating dynamic examination of complex joints such as the shoulder [1]. The US volumes are commonly acquired using 3D phased array probes and mechanically swept 3D probes [2]. The main limitations of 3D US are related to its limited field-of-view (FOV), which remains substantially smaller than other comprehensive medical imaging modalities such as CT and MRI. The US probe size, beamforming techniques and bony imaged structures constrain the volume size and depth. These FOV limitations hinder US examination and interpretation of musculoskeletal (MSK) structures [3].

Comprehensive 3D US mosaicking involves aligning or registering partially overlapping 3D US images of MSK landmarks to enable visualisation and quantitative measurement of an entire anatomical region and its local structures that are normally too large to be captured in a single US volume [4]. From a clinical perspective, this approach provides improved spatial context for musculoskeletal assessment, facilitating interpretation of complex joint anatomy and relationships between bony and soft-tissue structures [4] [5]. It also establishes a foundational real-time spatial framework that is a prerequisite for emerging applications such as US-based bone tracking and motion analysis when coupled with pre-acquired MRI or CT-based bone geometry [6]. Previous work demonstrated the feasibility of constructing a whole-shoulder 3D US mosaic using expert-conducted manual registration, yielding clinically viable visualisation of osseous and soft-tissue structures [5]. However, the approach required extensive manual effort (about 240h) by three experts for a single subject, limiting scalability, repeatability, and the ability to acquire sufficiently large datasets required for future real-time, data-driven deep-learning-based 3D US mosaicking [5].

3D US mosaicking is often performed by either pose estimation and/or image registration approaches [7]. Pose estimation techniques localise a US probe in space using a tracking system and align the acquired 3D US images by deducing their relative poses through calibration. The type of tracking system and calibration procedure have the most impact on estimation. Studies report on three common systems used to track US probes: optical, electromagnetic, and mechanical tracking systems [7]. The former two systems track the US probe via an attached sensor either optically (using multiple cameras) or electromagnetically. The limitation of each of these systems is that one requires a clear line of sight between the attached sensor and the tracking device, while the other introduces sensitivity to the metals in the electromagnetic field [8–12]. These limitations can make the data collection process challenging, especially when mosaicking large regions. On the other hand, mechanical systems have the US probe attached to the tip (end-effector) of an articulated robot arm. The end-effector pose is estimated by encoders located in each joint [7]. The main disadvantage of mechanical systems is that they can be cumbersome, and to date, no studies have investigated mechanically tracking 3D US probes for mosaicking purposes. Apart from the tracking system selection, the calibration approach also heavily impacts the performance of the pose estimation technique. Calibration transforms the pose of the tracked sensor/end-effector attached to the US probe to the pose of the acquired US volume. While there are numerous ways to determine that transformation, hand-eye calibration (HE) is one of the most recommended approaches [13]. At the time of reporting this study, nearly all 3D US calibration previous works pre-date 2009, and the average 3D US calibration reconstruction error reported in the literature was approximately 2–3 mm [13].

Another common 3D US mosaicking approach is image registration, which is either used independently or to refine the pose estimation volume alignment results, accounting for anatomical movements unregistered by the tracking system due, for example, to patient motion or respiration [10]. 3D US image (pairwise) registration is the alignment of two overlapping 3D US images based on mutual image content. The alignment of the two images may be performed automatically or manually. Expert-conducted manual US registration remains the most reliable approach and is regularly used as ground truth for validation of proposed automatic approaches [14–17]. This is despite being laborious, time-consuming, requiring experienced operators and unsuitable for extensive data or real-time applications [15]. Therefore, automatic 3D US image registration is an active area of research with numerous proposed algorithms and approaches. The most employed approach in the literature is correlation-based, intensity-based automatic image registration, which computes pairwise correlations in the image pairs [18]. Specifically, normalised cross-correlation (NCC) is often employed for its straightforwardness and independence from pre-defined image features despite its high computational cost [19]. As per the definition, since image registration is traditionally performed pairwise in mosaicking applications, where multiple 3D US images are involved, literature often decomposes the global alignment problem of mosaicking multiple volumes into a pairwise problem and employs 3D US image registration techniques [8,10–12,20]. This technique has been shown to lead to error propagation and suboptimal global alignment of the US volumes [21–24]. To address this issue, few studies have experimented with groupwise registration or simultaneous registration of multiple images to consider some valuable global information often discarded by pairwise approaches. This was either done monomodally (US-US) or multimodally (US-MRI) [5]. Novel automatic groupwise 3D US image registration algorithms, however, are mainly limited to phantom studies and their computational expense limits their use on a large number of volumes [4,24].

Deep-learning-based 3D mosaicking algorithms could be a promising alternative to currently used methods since they typically show state-of-the-art performance across a wide range of image analysis tasks. These methods can potentially improve the accuracy of the current traditional methods for mosaicking while addressing their limitations. They can offer real-time performance but remain presently unexplored. In particular, these techniques can enable real-time mosaicking of the anatomy in future applications where multiple transducers could acquire images/volumes simultaneously. The downside of these approaches is their requirement of high-quality big data for training. The challenge of obtaining such data is that pose estimation US mosaics often require further refinements; manual registration shows impractical scalability, automatic pairwise approaches lead to error propagation and automatic Groupwise approaches pose computation limitations. Furthermore, the performance of automatic approaches remains unchallenged on large numbers of partially overlapping volumes, ample distance coverage or structurally complex anatomical regions such as the shoulder [25].

In this study, we propose a hybrid workflow for large-extent 3D ultrasound mosaicking of the shoulder. The workflow uses mechanically tracked pose estimation to initialise alignment across all volumes, followed by hierarchical refinement using semi-automatic US–US registration and MRI-guided manual stabilisation to support globally coherent whole-shoulder reconstruction across multiple acquisition regions. The objectives are to (i) demonstrate the in-vivo feasibility of constructing continuous whole-shoulder 3D US mosaics from multi-region acquisitions in five healthy volunteers, (ii) quantify the extent to which a hybrid refinement approach improves reconstruction relative to pose initialisation alone and analyse the practical limits imposed by overlap and MRI–US pose variability, and (iii) evaluate post-processing/compounding strategies and derive practical acquisition and overlap requirements that enable scalable generation of high-quality mosaics for future automated, real-time, data-driven mosaicking approaches.

## 2. Materials and methods

### 2.1. Experimental setup

#### 2.1.1. Participants.

Five healthy volunteers were recruited from a previous study [26]. Three of the volunteers were male, and two were female. The mean and standard deviation for age was 25.0 ± 2.35 years, weight was 71.8 ± 14.72 kg, and height was 170.8 ± 6.86 cm. They had no history of shoulder operations or injuries. The ethics approval for the examinations, which took place from 09/11/2023 until 19/02/2024, was granted by the QUT Human Research Ethics Committee under the following number (No. 1700001110). Written informed consent was obtained from each participant (with at least one witness and no minors). 3D surface models of their respective clavicle, scapula and humerus bones were acquired from the same previous study [26].

#### 2.1.2. Acquisition systems: US, tracking system and MRI.

We acquired US volumes using a 3D linear mechanical probe. Specifically, we opted for the VL13−5 3D US probe, coupled with a Philips EPIQ7 US system (Philips Medical Systems, Andover, MA, USA) with a FOV of 38 mm × 30 degrees of sweep and a frequency range of 5–13 MHz. The US system parameters were set by a certified sonographer to a shoulder preset, with a penetration depth of 4 cm, wide imaging, XRES image processing and SonoCT compound imaging. The US volume resolution was (512 × 403 × 256) voxels with spacings of 0.1229 mm × 0.107 mm × 0.2405 mm.

The mechanical tracking system employed was the Franka Emika Panda robot, a 7-axis robot arm [27] with 850 mm reach, a 0.1 mm repeatability and an encoder resolution of 0.05 mm [27].

A corresponding shoulder and upper arm MRI of the same subject was also already available from a previous study [26] using a 3-Tesla MR scanner (Ingenia, Koninklijke Philips N.V., Best, The Netherlands) at voxel dimensions of 0.4 × 0.4 × 0.8 mm. The MRI was performed with the subject supinely, using cushions under the shoulder, causing slight anterior arm rotation. We specifically utilized the bone segmentations derived from the MRI data including the humerus, scapula and clavicle.

#### 2.1.3. Acquisition system calibration.

After securely mounting 3D US probe onto the robot end-effector using a custom-designed probe holder, the transformation ($\backslash \overset{P}{\underset{I}{(}}X\;$) from the robot end-effector to the US images was determined. This was implemented using HE calibration proposed by [28] for the calibration of tracked 3D US through the alignment of 3D US structures. The calibration was based on a closed-form solution implemented by [29] which solves for translations and quaternion rotations of the transformation separately. The calibration was performed on a breast phantom by aligning its insertions in 60 partially overlapping volumes involving diverse probe rotations and translations. The movements involved in the calibration acquisition protocol of the phantom are depicted in S1 Fig. **US Calibration Protocol**.

The acquired US calibration volumes were imported and manually aligned in (ImFusion GmbH, München, Bayern, Germany). The 3D transformation of each US volume after they have been aligned in the software was recorded as A. The corresponding robot end-effector poses relative to the robot base frame were recorded using a custom-written Python script and labelled Ts. The displacement between consecutive poses $T_{s\text{1}}$ and $T_{s\text{2}}$ was labelled as B and calculated using the formula provided by [28]:


$$\begin{array}{c} B={T_{s\text{2}}}^{-\text{1}}{\;T}_{s\text{1}} \end{array}$$

The hand-eye calibration equation that finds the transformation ($\backslash \overset{P}{\underset{I}{(}}X\;$) between the US frame and the robot base frame was defined by:


$$\begin{array}{c} \overset{P}{\underset{I}{A}}X\;\overset{P}{\underset{I}{=}}X\;B \end{array}$$

The US calibration error was evaluated using the reconstruction accuracy metric [28] by mapping test points in the US volumes to both the US frame using $\backslash \overset{P}{\underset{I}{(}}X\;$ and the true space (in manually aligned volumes). The root mean square error (RMS) distance between the US frame points and corresponding true points was computed.

#### 2.1.4. Sonography protocol.

**Fig 1:**
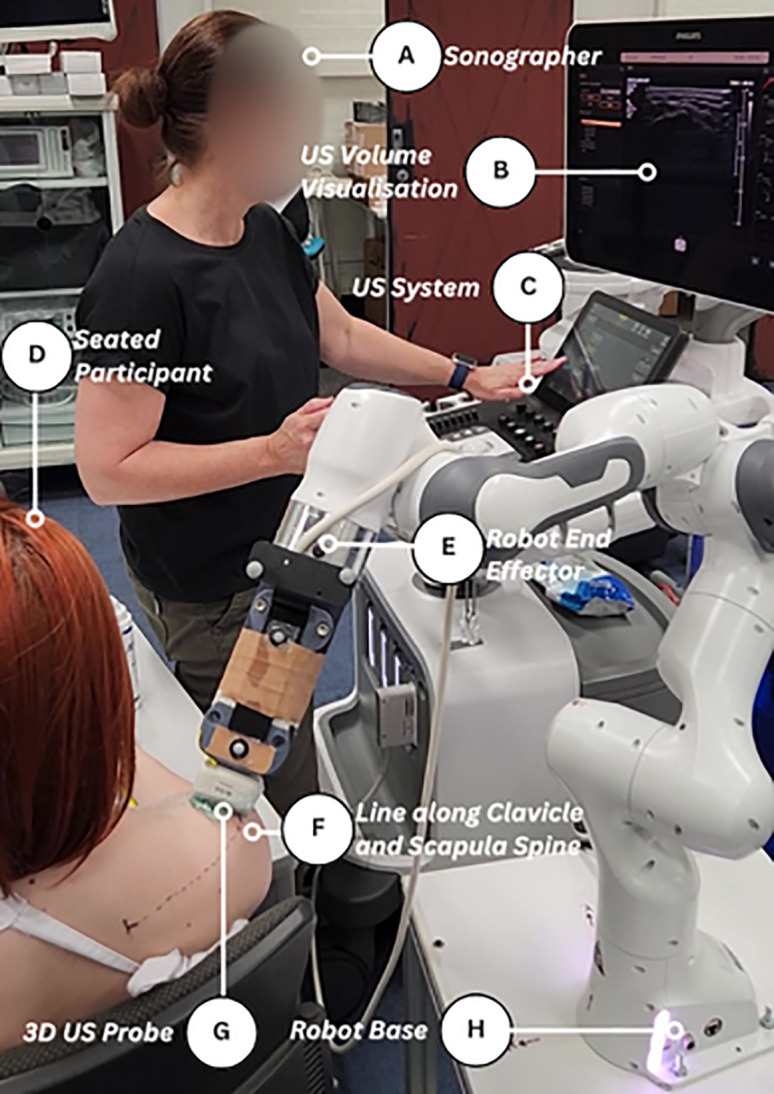
Sonography experiment setup. The US probe is rigidly mounted to the robot end effector to ensure stable volumetric acquisition while the participant remains seated. Key system components and anatomical guidance used to direct probe trajectories along the clavicle and scapular spine are indicated. The figure illustrates setup of the robotic tracking system, participant, US hardware, and dotted line used to guide the sonographers throughout the acquisition protocol.

The sonography experiments were conducted by two experienced, registered sonographers. Fig 1 illustrates the acquisition system setup used during the experiments. Volunteers were seated with the back and forearms supported, and right shoulder exposed. Each volunteer underwent one acquisition with the forearm resting on the table at shoulder width (approximately 90 deg relative to the torso), with two volunteers additionally scanned at 45 deg proximal arm rotation to introduce controlled pose variability.

The shoulders were divided into five regions (series 1–5 respectively): anteroinferior (IA), anterosuperior, posteroinferior (IP), posterosuperior and superior. This acquisition order was selected following preliminary testing to minimise probe repositioning and participant movement. Prior to scanning, key anatomical landmarks were identified using a standard 2D linear probe and marked to guide probe trajectories. These landmarks included the medial and lateral clavicle, scapular spine, lateral humerus, and acromioclavicular joint (ACJ). 3D US volumes were acquired following predefined trajectories for each region, maintaining an approximate spacing of 3 cm between consecutive volumes. The scanning routine, including anatomical start and end points, probe paths, probe orientation, and number of volumes per region, is summarised in Table 1. The detailed probe–anatomy relationship during acquisition is illustrated in S2 Fig. **Sonography Protocol**.

**Table 1:** Scanning Routine: The table discusses regions scanned, probe trajectory and number of volumes scanned. It also mentions the approximate number of US volumes acquired per trial and indicates which half of the US probe was aligned with the guiding structure (probe path) during the imaging of each region (probe orientation relative to the path).

Scanning Routine					
Regions	Starting Structure	Destination	Probe Path	Probe Orientation Relative to Path	Number of Volumes
Anteroinferior	Medial clavicle	Lateral Humerus	Clavicle line	Top of the probe	12
Anterosuperior	Medial clavicle	ACJ	Clavicle line	Bottom of the probe	8
Posteroinferior	Medial scapula	Lateral Humerus	Scapula spine	Top of the probe	10
Posterosuperior	Medial scapula	ACJ	Scapula spine	Bottom of the probe	10
Superior	Superior remnants	ACJ	Lateral shoulder	Centre of the probe	5

Volumes were recorded when the target anatomy was clearly visualised in the US image. Each volume was synchronously captured with the corresponding robot arm pose during a 5 s sweep, while minimising probe pressure to avoid subject or probe displacement.

During the scan, whenever the sonographer decided that the respective shoulder anatomy was clearly demonstrated in the US view on the US machine, the US volume and its corresponding robot arm pose were recorded. The robot arm acted as a rig, securing the probe during acquisitions for successful 5s sweeps and an accurate corresponding robot arm pose. The sonographer avoided excessively pressing the probe against scanned anatomy to prevent displacing the volunteer and/or the probe with respect to the robot arm.

### 2.2. Reconstruction protocol

Each trial was reconstructed from approximately 45 partially overlapping US volumes using two approaches:

(i) pose estimation only
(ii) a hybrid reconstruction framework integrating pose estimation with monomodal and multimodal registration

The entire mosaicking protocol was performed using an iterative, consensus-based workflow involving three experienced operators, with final adjudication by a registered sonographer when discrepancies arose.

#### 2.2.1. Overview.

**Fig 2:**
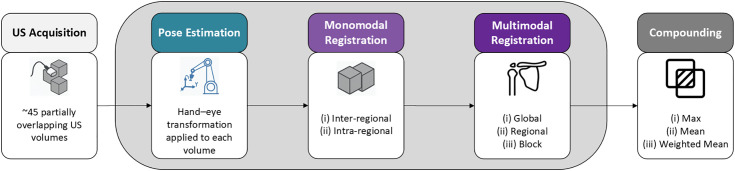
3D US Hybrid reconstruction framework. Partially overlapping US volumes are first positioned using pose-based alignment obtained from robot tracking and hand–eye calibration. Alignment is subsequently semi-automatically refined using monomodal US–US registration followed by multimodal US–MRI registration performed at global, regional, and block levels. The resulting refined alignment is then used for regional and global compounding to generate the final US reconstructions.

The reconstruction workflow is summarised in Fig 2. Pose-based alignment obtained from robot tracking and hand–eye calibration was used to initialise the spatial relationship across all US volumes. This initial solution was subsequently refined through two stages:

1. **Monomodal refinement (US–US):** semi-automatic registration to improve local anatomical continuity within and between acquisition regions.
2. **Multimodal refinement (US–MRI guidance):** manual registration guided by subject-specific MRI bone segmentations to improve global anatomical coherence.

Registration was performed hierarchically at global, regional, and block (four-volume) levels, with pose anchoring strategies applied to preserve established relative alignments. We finally compound the aligned volumes in the pose estimation and hybrid solutions into US mosaics.

#### 2.2.2. Pose estimation.

Following calibration, all US volumes were positioned in a common reference frame by applying the hand–eye transformation $\backslash \overset{P}{\underset{I}{(}}X\;$ to the corresponding robot end-effector pose ${\;T}_{s}$. Volumes were converted into a MetaImage MetaHeader (MHD) file type using a custom-written MATLAB script and then imported into ImFusion for visualisation and further processing. This pose-based alignment served as a coarse but consistent initial estimate for subsequent refinement.

#### 2.2.3 Monomodal registration.

Monomodal refinement aimed to improve local alignment between overlapping US volumes while maintaining global consistency. Registration was performed semi-automatically using a fast normalised cross-correlation (NCC) similarity metric optimised with the Nelder–Mead simplex algorithm, combined with expert-guided manual adjustments. Further details on the semi-automatic algorithm are in S1 Text. **Semi-Automatic Approach Details**. The Registration was conducted in two stages:

- **Intra-regional refinement:** overlapping volumes within the same acquisition region were aligned sequentially, with pose anchoring applied to preserve previously established relative transformations.
- **Inter-regional refinement:** boundary volumes near the acromioclavicular joint were reviewed to establish or refine spatial relationships between regions. Where monomodal alignment was unreliable due to limited overlap, pose-based relationships were retained.

**Fig 3:**
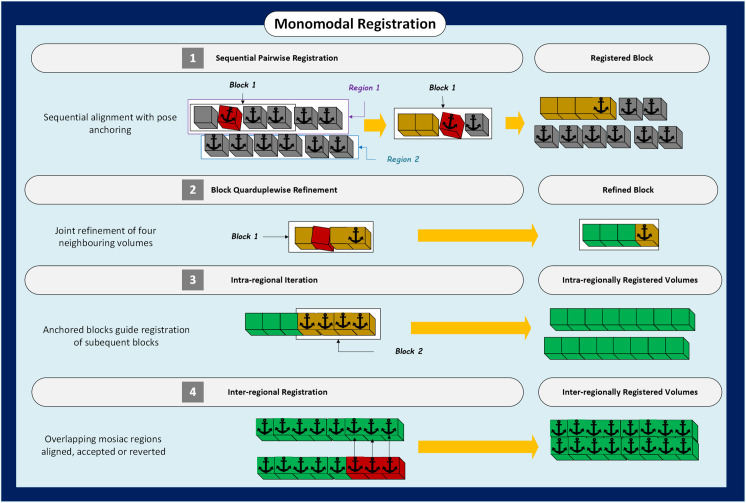
Schematic overview of the semi-automatic monomodal (US–US) registration process used to refine pose-based alignment within and between acquisition regions. Individual blocks represent 3D US volumes, with anchor symbols indicating pose-anchored volumes whose relative transformations are preserved during refinement. Step 1 (Sequential pairwise registration): Overlapping volumes are aligned sequentially within each region while previously registered neighbours remain anchored, preserving established local and global relationships. Step 2 (Block quadruplewise refinement): Groups of four neighbouring volumes are jointly refined to reduce cumulative pairwise registration error and improve local anatomical coherence. Step 3 (Intra-regional iteration): Refined blocks are used as anchors to guide registration of subsequent blocks, enabling consistent propagation of alignment across the region. Step 4 (Inter-regional registration): Overlapping volumes from adjacent regions are assessed to establish inter-regional continuity; alignments are accepted, refined, or reverted to pose-based relationships when overlap is insufficient. Colours denote registration status: unregistered (grey), actively refined (red), registered blocks (yellow), and final intra- or inter-regionally registered volumes (green). The workflow prioritises preservation of global consistency while improving local anatomical alignment.

The full registration workflow is summarised in Fig 3. Intra-regional refinement followed a structured block-wise strategy. Volumes were first aligned sequentially in a pairwise manner while anchoring previously registered neighbours (Step 1). Groups of four neighbouring volumes were then jointly refined to reduce cumulative pairwise registration error and improve local anatomical coherence (Step 2). These refined blocks subsequently served as anchors to guide registration of neighbouring blocks within the same region, enabling consistent propagation of alignment across the region (Step 3). Finally, overlapping volumes from adjacent regions were assessed to establish inter-regional continuity (Step 4). Depending on the quality and extent of overlap, inter-regional alignments were accepted, refined, or reverted to the initial pose-based relationship to avoid introducing global inconsistencies. Overall, monomodal refinement primarily improved anatomical continuity within regions and reduced local misalignments while preserving a globally coherent reconstruction.

The multimodal registration phase incorporated the patient-specific MRI bony segmentation to further refine the global alignment of the monomodally registered 3D US mosaic. The STL models of the humerus, scapula and clavicle bones were imported into ImFusion, combined and converted into a single label map, visualised alongside the US volumes.

#### 2.2.4. Multimodal registration.

To further improve anatomical coherence of the US shoulder reconstruction, a multimodal refinement stage was introduced using patient-specific MRI bone segmentations as anatomical guidance. The aim was to reduce residual global inconsistencies arising from limited overlap between shoulder acquisition regions and cumulative registration error.

STL segmentations of the humerus, scapula, and clavicle derived from MRI were imported and visualised alongside the monomodally registered US mosaic. Due to differences in shoulder pose between US and MRI acquisitions (Section 2.1.2), MRI data were not used as a strict registration target. Instead, they served as a visual reference to guide refinement of global and inter-regional relationships without compromising established monomodal alignments.

Multimodal refinement was performed hierarchically in three stages:

- **Global coarse registration**: the entire US mosaic was rigidly aligned to the MRI bone segmentation to establish an overall anatomical correspondence while preserving all monomodal relative transformations.
- **Regional refinement**: individual acquisition regions were subsequently refined relative to the MRI guidance to improve consistency between neighbouring regions.
- **Block-level refinement**: small sets of neighbouring volumes were locally refined to improve anatomical coherence while maintaining both monomodal continuity and global alignment.

Throughout this process, pose anchoring was applied to prevent disruption of previously established alignments, and priority was given to bony structures due to their non-deformable nature. In cases of conflict between monomodal alignment and multimodal correspondence, monomodal consistency was preserved to maintain a globally coherent reconstruction.

This multimodal stage primarily served to stabilise global anatomy and inter-regional relationships, complementing the local accuracy achieved through monomodal refinement.

#### 2.2.5. Compounding.

Following alignment protocol, aligned US volumes were compounded to generate regional and global shoulder mosaics. Compounding was performed separately for the five acquisition regions (regional mosaics) and for the whole shoulder (global mosaic).

Regional mosaics were directly generated to preserve spatial resolution, while global mosaics were downsampled to reduce computational cost associated with resampling and interpolation of large, high-resolution datasets. Further downsampling details are discussed in S2 Text. **Downsampling**.

Three compounding strategies were investigated: maximum (Yao et al., 2009), mean (Yao et al., 2009) and weighted mean voxel intensity values [30]. Weighted mean or averaging is the implementation where voxel intensities from different volumes merge through weights determined by relative quality or other confidence measures [31]. Similar to [32], the weighting measure we opted for with overlapping intensities is the Euclidean distance to each volume centre.

Unless otherwise stated, maximum compounding was used for primary visualisation and evaluation to enable direct comparison with existing extended FOV US literature. A comparative analysis of compounding strategies is presented in **Section 3.4**.

### 2.3. Reconstruction evaluation

Quantitative evaluation of in vivo US mosaicking is challenging due to the absence of ground-truth anatomical alignment. Consistent with prior work, reconstruction accuracy was therefore assessed comparatively, evaluating improvements achieved through the hybrid reconstruction framework relative to pose estimation alone [10].

Evaluation focused on the inferior humerus, selected for its size, non-deformable nature, asymmetry, and consistent visibility across overlapping volumes. Analyses were performed both regionally, on the IA and IP regions, and globally, on the whole-shoulder mosaic.

#### 2.3.1. Improvement in monomodal alignment.

**Fig 4:**
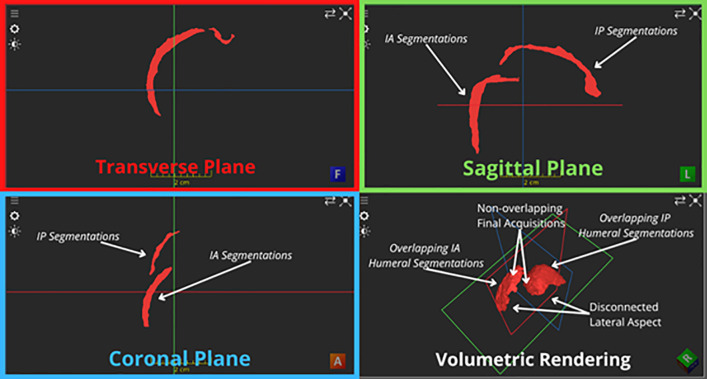
Monomodal humeral segmentation visualisation example.

Improvement in monomodal alignment involved segmenting the humerus from the final five overlapping volumes in each inferior region (IA and IP), where sufficient anatomical coverage was available (lateral humerus). Segmentations were transformed using the estimated poses obtained from pose estimation and hybrid reconstruction, respectively. Details of the segmentation procedure and parameter selection are provided in S3 Text. **Segmentation details**. Fig 4 shows the segmentation visualisation of an example trial using the hybrid method.

Alignment between neighbouring humeral segmentations was quantified using the Dice similarity coefficient [33] metric, which is defined by:


$$\begin{array}{c} \frac{\text{2}|A\cap B|}{\left|A|+|B\right|}*\text{100} \end{array}$$

Where |A| and |B| are the number of voxels in each respective humerus segmentation and |A∩B| is the number of overlapping voxels between them. Higher dice coefficients indicate better overlap, with 100 representing perfect overlap and zero representing no overlap. Dice scores were used comparatively to evaluate relative improvements in alignment, acknowledging that partial anatomical overlap inherently limits absolute Dice values. Intra- and inter-regional average Dice coefficients were reported.

#### 2.3.2. Effect of multimodal pose variability.

To quantify the impact of differences in shoulder pose between US and MRI acquisitions, multimodal alignment was evaluated by comparing hybrid US humeral segmentations with corresponding MRI counterpart.

**Fig 5:**
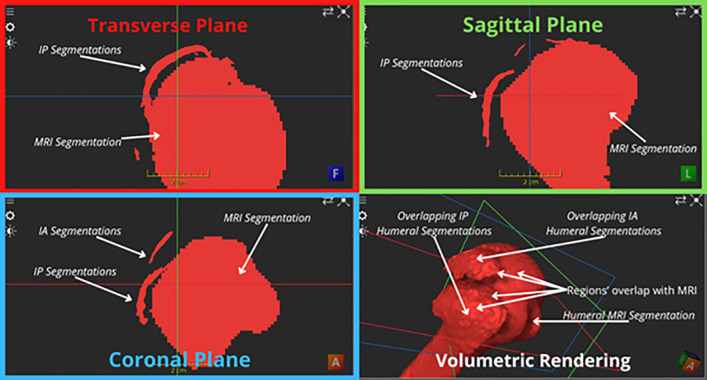
Multimodal humeral segmentation visualisation example.

Fig 5 shows an example visualisation of the environment after introducing the MRI humeral segmentation.

Rather than assessing absolute multimodal registration accuracy, which is confounded by pose mismatch, evaluation focused on the **relative overlap** between US and MRI segmentations. This metric reflects the proportion of the US segmentation that spatially overlaps the MRI segmentation and serves as an indicator of the practical limits of multimodal refinement under differing acquisition poses. Relative overlap was defined as follows, where A and B are the number of voxels in the US and MRI segmentation, respectively:


$$\begin{array}{c} \frac{\left|A\cap B\right|}{\left|A\right|}*\text{100} \end{array}$$

Intra- and inter-regional overlap values were computed and reported across trials.

## 3. Results

The sonography protocol was successfully completed for all seven trials (PA01–07), with each shoulder acquisition requiring approximately 45–60 minutes, including subject positioning and volume acquisition. The mean ± SD calibration RMS error was 1.3 ± 0.42 mm (computed across the two trials, RMSE = 1.0 mm and 1.6 mm).

Reconstruction time differed substantially between methods. Pose estimation–based reconstruction was completed immediately following acquisition, whereas the hybrid mosaicking framework required approximately **32 hours per trial**, reflecting the iterative monomodal and multimodal refinement process performed by experienced operators.

Across all seven trials, both pose estimation and hybrid mosaicking produced continuous shoulder reconstructions without notable gaps. However, clear differences were observed in anatomical continuity, alignment accuracy, and robustness to limited overlap between acquisition regions.

### 3.1. Visual improvement in reconstruction accuracy

**Fig 6:**
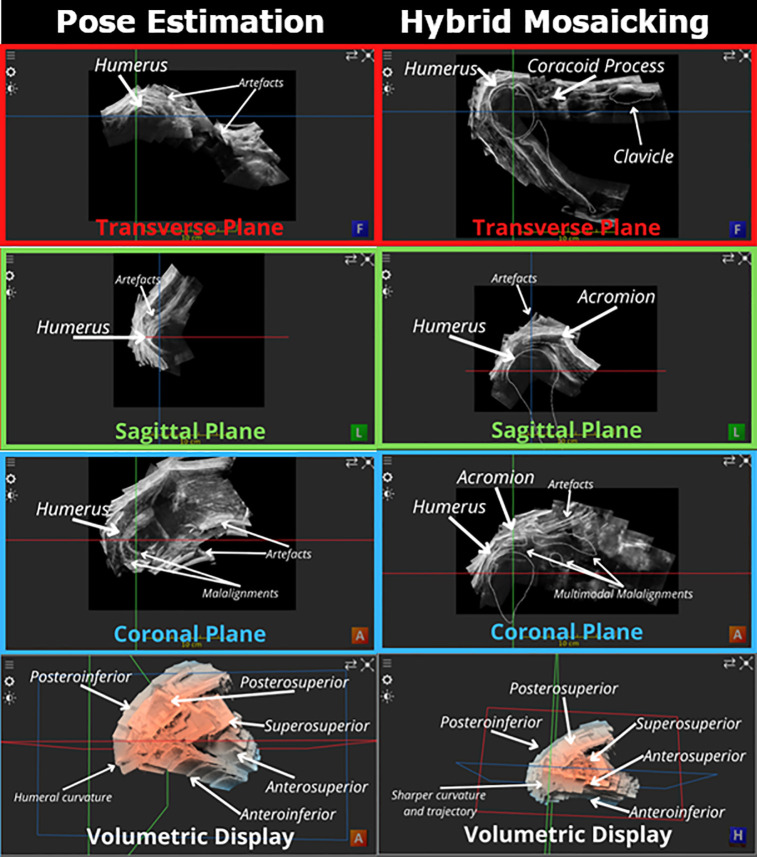
Global US shoulder reconstruction using pose estimation and hybrid mosaicking. Representative global 3D US shoulder reconstructions from one trial using pose estimation (left) and hybrid mosaicking (right). Reconstructions are shown in three orthogonal planes (transverse, sagittal, coronal) and volumetric rendering. The pose-based reconstruction broadly follows the expected shoulder geometry but exhibits visible discontinuities and artefact accumulation. The hybrid reconstruction demonstrates improved local anatomical continuity and reduced artefacts, enabling clearer visualisation of musculoskeletal structures (humerus, clavicle, coracoid process and acromion), while remaining constrained by limited inter-regional overlap between acquisition regions.

**Fig 7:**
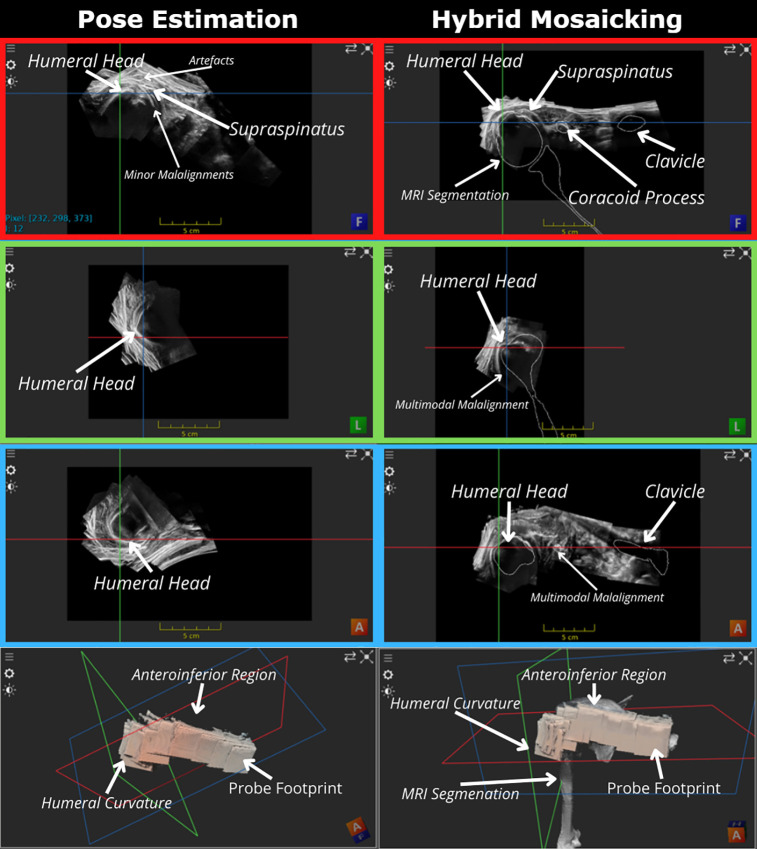
Regional US shoulder reconstruction using pose estimation and hybrid mosaicking. Representative regional 3D US reconstructions of the inferior shoulder region using pose estimation (left) and hybrid mosaicking (right). Reconstructions are shown in three orthogonal planes and volumetric rendering. Regional mosaics comprise fewer volumes than global reconstructions, resulting in smoother transitions, fewer discontinuities, and clearer delineation of osseous and soft-tissue structures for both methods. Hybrid mosaicking shows altered representation of humeral curvature relative to pose estimation, including instances of unrealistically sharp curvature.

Qualitative comparison of pose estimation and hybrid mosaicking is shown in Fig 6 **(global reconstruction)** and Fig 7 **(regional reconstruction)**. The visual global reconstruction results for the **pose estimation** and **hybrid approach** across all trials are illustrated in S3 Fig. **Global Visual Results Pose Estimation** and S4 Fig. **Global Visual Results Hybrid**.

Across all trials, **intra-regional overlap** was consistently higher than **inter-regional overlap**. On average, every three intra-regional volumes shared a mutual overlap, whereas structural overlap between neighbouring acquisition regions was substantially lower and often limited to small boundary areas and near the acromioclavicular joint. This imbalance strongly influenced reconstruction behaviour at both regional and global scales.

Trials PA001A, PA002A and PA002B qualitatively exhibited the most accurate shoulder reconstruction using both mosaicking approaches. PA001B and PA003A showed the lowest humeral demonstration in the pose estimation-based solution and experienced the most substantial qualitative improvement using the hybrid method in anatomical demonstration.

At the **global level**, pose estimation produced reconstructions that broadly followed the expected shoulder geometry and probe footprint but frequently exhibited local artefacts, discontinuities, and misalignments, particularly in regions of high curvature and at inter-regional boundaries. Hybrid mosaicking reduced artefact accumulation and improved anatomical continuity, enabling clearer visualisation of additional musculoskeletal structures, including the clavicle, coracoid process, and supraspinatus tendon. Nevertheless, limited inter-regional overlap constrained refinement in some regions, leading to residual misalignments despite overall improvement.

At the **regional level**, both pose estimation and hybrid mosaicking exhibited improved visual clarity compared to global reconstructions. Regional mosaics comprised approximately **12 volumes**, compared to **~45 volumes** in global reconstructions, and consistently showed clearer delineation of osseous and soft-tissue structures, including the humeral head and supraspinatus. The mosaicked regions appeared smoother and more continuous, with fewer visible discontinuities and artefacts. This trend was observed for both reconstruction methods and coincided with the reduced number of compounded volumes in regional mosaics. Notably, hybrid mosaicking often produced a more sharply defined humeral curvature than pose estimation, which in some cases appeared unrealistically sharp relative to expected anatomical geometry.

Taken together, visual assessment showed that hybrid mosaicking improved local anatomical continuity and reduced artefact accumulation relative to pose estimation, while remaining constrained by acquisition overlap and exhibiting method-specific artefacts at both regional and global scales.

### 3.2. Quantitative improvement in reconstruction accuracy

Quantitative evaluation of reconstruction accuracy is summarised in Table 2, which reports regional Dice similarity coefficients for IA and IP regions, as well as global connectivity characteristics, for pose estimation and hybrid mosaicking.

**Table 2:** Regional and global dice coefficients and overlap details.

	Regional Dice Coefficients				Global Dice Coefficients and Overlap			
Label	IA		IP		Final Regional Volumes		Lateral Aspect Connection	
	(P)	(H)	(P)	(H)	(P)	(H)	(P)	(H)
PA001A	11.17%	16.71%	21.79%	29.01%	0.00%	0.00%	Connected	Disconnected
PA001B	20.81%	39.62%	1.25%	32.67%	0.00%	0.00%	Connected	Connected
PA002A	26.82%	53.20%	10.03%	41.85%	17.73%	0.00%	Connected	Disconnected
PA002B	12.28%	51.61%	10.44%	10.34%	0.00%	0.00%	Connected	Disconnected
PA003A	0.12%	21.90%	4.88%	53.55%	0.00%	0.00%	Disconnected	Disconnected
PA004A	14.86%	7.15%	3.58%	3.58%	0.33%	0.00%	Connected	Connected
PA005A	38.14%	49.77%	2.84%	2.84%	0.00%	0.00%	Connected	Disconnected
Overall	17.74%	34.28%	7.83%	24.83%	2.58%	0.00%	Connected	Disconnected

Across all trials, hybrid mosaicking produced higher regional Dice coefficients than pose estimation in both inferior regions. Averaged across trials, Dice coefficients increased from **17.74% to 34.28%** in the IA region and from **7.83% to 24.83%** in the IP region when using the hybrid approach.

Direct overlap between the final volumes of the IA and IP regions was generally limited. Using pose estimation, the lateral aspect of the inferior shoulder was connected in all trials except **PA003A**, resulting in a small but non-zero final-volume overlap (mean **2.58%**). In contrast, hybrid mosaicking resulted in disconnection of the final inferior volumes in most trials, with direct overlap preserved in only two cases.

These results indicate that while hybrid mosaicking improved intra-regional alignment between overlapping volumes, inter-regional connectivity remained strongly dependent on the spatial overlap available at regional boundaries.

### 3.3. Effect of varying multimodal pose

Here, we show the effect of the varying multimodal shoulder pose on the reconstruction results. Quantitative multimodal alignment results between hybrid US mosaics and MRI-derived humeral segmentations are summarised in Table 3, which reports relative overlap values for the IA, IP and global reconstructions.

**Table 3:** Average relative overlap of the US humeral segmentations w.r.t. the MRI counterpart in the IA and IP regions as well as the whole mosaic reconstructed using the hybrid method.

Label	Regional IA (H)	Regional IP (H)	Global Mosaic (H)
PA001A	34.89%	0.00%	17.45%
PA001B	0.00%	0.00%	0.00%
PA002A	7.98%	43.35%	25.67%
PA002B	45.16%	12.77%	28.96%
PA003A	0.00%	1.16%	0.58%
PA004A	25.09%	36.39%	29.33%
PA005A	0.00%	39.75%	19.88%
Overall	16.16%	19.06%	17.41%

Across all trials, multimodal overlap between US and MRI humeral segmentations was limited. Averaged across trials, relative overlap was **16.16%** in the IA region, **19.06%** in the IP region, and **17.41%** for the global mosaic. The highest multimodal alignment was observed in PA002A, PA002B, and PA004, with an overlap of 25.67%, 28.96% and 29.33%, respectively. Minimal or no global overlap with the corresponding humeral MRI was observed in PA001B and PA003A (0.00% and 0.58%, respectively).

Substantial regional asymmetry was observed within individual trials. For example, **PA002A** exhibited higher overlap in the IP region (**43.35%**) than in the IA region (**7.98%**), while **PA002B** showed higher overlap in the IA region (**45.16%**) than in the IP region (**12.77%**). Similar regional imbalance was observed in **PA001A** (IA **34.89%**, IP **0.00%**) and **PA005A** (IA **0.00%**, IP **39.75%**), despite comparable global overlap values.

### 3.4. Comparison of compounding techniques

**Fig 8:**
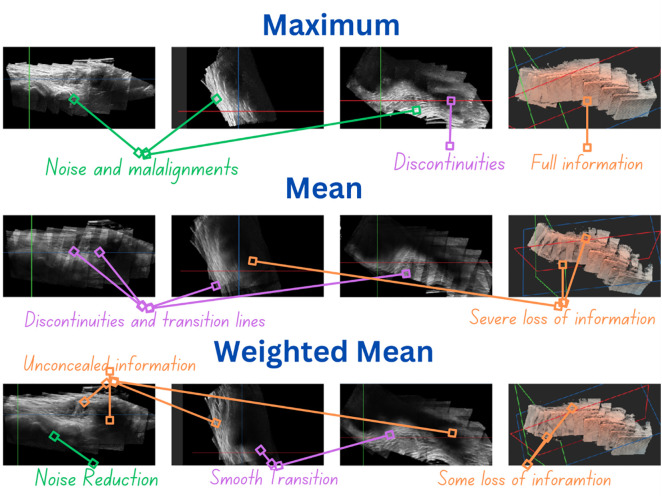
Comparison of reconstruction performance using Maximum, Mean, and Weighted Mean compounding (top, middle and bottom, respectively) on the IA region of trial PA001A. The four mosaic views showed (left to right) are transverse, sagittal, coronal and volumetric.

**Fig 9:**
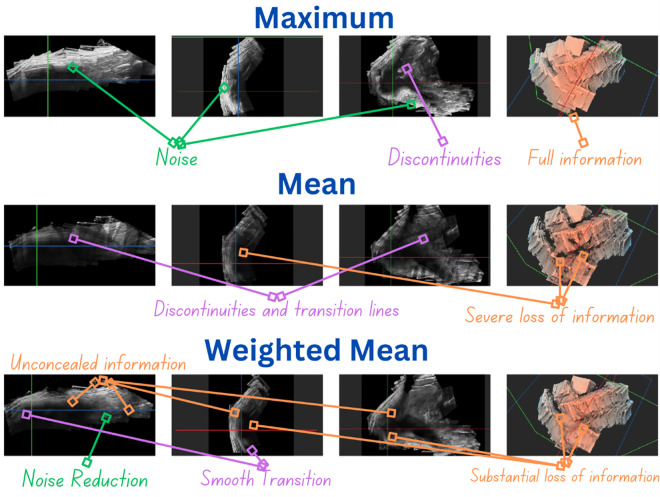
Comparison of reconstruction performance using Maximum, Mean, and Weighted Mean compounding on the global mosaic of trial PA001A. The four mosaic views illustrated (left to right) are transverse, sagittal, coronal and volumetric.

The effect of compounding strategy on reconstruction quality is illustrated in Fig 8 (regional reconstructions) and Fig 9 (global reconstructions), which compare maximum, mean, and weighted mean compounding for representative trials. We compare the reconstructions in terms of noise, anatomical alignment, structural smoothness, volume transition continuity and information retention.

At the **regional** level (Fig 8), where substantial overlap existed between adjacent volumes:

- **Maximum compounding** preserved all contributing voxel information but amplified noise and misalignment artefacts, resulting in visible discontinuities where individual volumes were imperfectly aligned.
- **Mean compounding** reduced noise but suppressed anatomical detail, particularly in regions with limited overlap, leading to loss of clearly identifiable landmarks.
- **Weighted mean compounding** produced smoother transitions between volumes and reduced artefact accumulation while maintaining visual continuity of osseous and soft-tissue structures within overlapping regions.

At the **global** level (Fig 9), where inter-regional overlap was limited:

- **Mean and weighted mean compounding** resulted in substantial loss of anatomical information in non-overlapping regions, producing fragmented or incomplete shoulder reconstructions.
- **Maximum compounding** preserved structural continuity across the full shoulder extent despite increased artefact visibility and was the only compounding strategy to yield complete global reconstructions under the observed overlap conditions.

## 4. Discussion

This study demonstrated the feasibility of reconstructing the entire shoulder of five participants as a single, coherent 3D US visualisation (mosaic) using a novel hybrid mosaicking workflow. Beyond feasibility, the key contribution is a multimodal reference reconstruction pipeline designed to maximise global coherence under realistic in vivo overlap and motion constraints, thereby enabling generation of the highest-quality mosaics needed to train and benchmark future automated, real-time mosaicking approaches. In workflows where MRI/CT is already acquired, the same dataset can be repurposed as a subject-specific anatomical prior and quality-control benchmark for US mosaic construction instead of in vivo ground truth, while preserving the longer-term goal of reducing external diagnostic modality dependence through improved overlap and automation. Two certified sonographers successfully imaged the shoulder as a whole of five volunteers using a mechanically tracked 3D US probe, with the arm rested in a natural shoulder-width position at 90 deg flexion, and additional trials acquired at 45 deg proximally rotation. Each of the seven total shoulder trials comprised approximately 45 US volumes, broken down into five anatomical regions (5–12 volumes each): IA, IP, SA, SP and SS. We compared two mosaicking strategies: pose estimation using mechanical tracking and hand-eye calibration, and a hybrid approach combining pose estimation with semi-automatic monomodal registration and MRI-guided multimodal refinement. Reconstruction accuracy was evaluated both visually and quantitatively, at regional and global scales.

For the **pose-based reconstruction**, qualitative analysis demonstrated anatomically plausible shoulder renderings consistent with the sonography protocol footprint and without notable gaps. However, substantial malalignments, noise and artefacts were present, particularly at inter-regional boundaries, suggesting a need for refinement and discouraging reliance on pose estimation alone. Intra-regional overlap was generally sufficient, with approximately every three adjacent volumes sharing a mutual overlap, and higher volume density observed near the ACJ. In contrast, inter-regional overlap was often limited outside this region. Quantitative results confirmed this pattern: while substantial partial overlap existed between adjacent intra-regional volumes (DnC IA: 17.74% and DnC IP: 7.83%), direct overlap between the final inferior volumes was absent in 5 of the 7 trials (DnC: 0%). Nevertheless, limited inter-regional overlap was still visually observed near the ACJ via neighbouring inferior volumes, rather than through direct overlap of the terminal volumes. These analyses suggested that in some cases, while the sonographer protocol captured the guiding anatomical structures in all regions, different sides of the structures may have been acquired during these experiments with little overlap of mutual parts of the structures themselves. This limited inter-regional overlap therefore emerged as a primary constraint on the refinement process.

For the **hybrid-based reconstruction**, the proposed workflow produced measurable improvements in reconstruction accuracy at both qualitative and quantitative levels. Visually, hybrid refinement reduced malalignments, artefacts, and discontinuities while enhancing the delineation of musculoskeletal structures (e.g., humerus, clavicle, coracoid process and acromion) without compromising overall shoulder geometry. These improvements were most apparent in regions with sufficient overlap (intra-regional acquisitions), where smoother transitions and improved anatomical continuity were consistently observed. Quantitatively, hybrid mosaicking increased intra-regional Dice similarity coefficients from 17.74% to 34.28% in the inferior anterior region and from 7.83% to 24.83% in the inferior posterior region, corresponding to an average improvement of approximately 17% in local overlap. These gains reflect the ability of the hybrid strategy to mitigate cumulative pairwise registration error. However, global inter-regional connectivity remained strongly dependent on available overlap at regional boundaries, with direct lateral humeral overlap preserved in only two trials. Together, these results show that while hybrid refinement substantially improves local anatomical alignment beyond pose estimation alone, achievable global reconstruction accuracy remains constrained by sparse inter-regional overlap, patient motion, and MRI pose variability.

**Mechanistically**, hybrid refinement behaved as a hierarchical, constraint-balancing process in which what could be improved was largely dictated by overlap, rotational offsets, and pose consistency. Semi-automatic monomodal registration efficiently stabilised intra-regional alignment when mutual overlap was clear: the automatic component primarily accelerated convergence, while expert guidance remained essential in ambiguous cases, and large relative rotations (notably near the lateral humerus) consistently proved more challenging than translational offsets. Pose anchoring and groupwise inspection helped limit error propagation and improved intra-regional consistency with a practical refinement time of ~9 h per trial; however, under sparse inter-regional overlap and occasional volunteer motion, purely image-based inter-regional refinement was often unreliable and was therefore applied conservatively, with boundary volumes frequently reverted to pose-based estimates to preserve global coherence. MRI-guided multimodal refinement was then most valuable as a global stabiliser and plausibility check to establish inter-regional relationships and detect locally plausible but globally implausible configurations, but its corrective capacity was fundamentally limited by MRI–US pose mismatch; region- and block-level adjustments often revealed competing monomodal and multimodal constraints, requiring prioritisation of monomodal continuity and bony alignment over strict US–MRI correspondence. This explains why multimodal refinement was the most time-consuming stage (~24 h per trial) and why strict rigid multimodal registration was not feasible under typical acquisition conditions (effective discrepancies up to ~82%), providing a clear basis for the comparisons and limitations discussed below.

**Evaluation of post-processing and compounding strategies** further demonstrated that compounding behaviour is strongly scale-dependent and intrinsically linked to acquisition overlap. At the regional level, where adjacent volumes exhibited substantial mutual overlap, weighted mean compounding produced the most visually balanced mosaics, reducing artefact accumulation while preserving anatomical detail and continuity. In contrast, at the global scale, inter-regional overlap was frequently sparse or absent, rendering mean and weighted mean compounding unsuitable due to suppression of non-overlapping anatomical content and fragmentation of the reconstruction. Under these conditions, maximum compounding remained the only strategy capable of preserving complete shoulder anatomy, albeit at the cost of increased noise and visible artefacts. Overall, compounding performance was governed primarily by overlap density, exposing a scale-dependent trade-off between visual smoothness and anatomical completeness. Accordingly, compounding strategy selection should be regarded as a core design consideration for scalable mosaicking rather than a post-processing choice.

There are several strengths and drawbacks in our mosaicking approach **compared to similar previous reports** by *Poon et al.* [10]. The differences in our findings may mainly be attributed to variations in experiment type (in-vitro fetus vs in-vivo shoulder), pose estimation tracking system (optical tracking vs mechanical tracking), acquisition system (low frequency phased array vs high-frequency linear array probe), calibration procedure (stylus vs hand-eye), mosaicking extent (10 vs 45 volumes) and registration approach (automatic rigid/non-rigid vs semi-automatic hybrid registration). In terms of our approach’s strengths, our pose estimation showed better average calibration errors (1.2 mm vs 1.75 mm), possibly due to acquiring more calibration volumes (20 vs 60 volumes), higher resolution imaging equipment (max probe frequency 8MHz vs 13MHz) and more stable probe during acquisition (supported by fixed robot arm vs manually operated optically tracked probe). The visible malalignments in their in-vitro fetus phantom were comparable to our in-vivo pose estimation reconstruction. For mosaicking refinement, our hybrid approach showed significantly better reconstruction accuracy improvements than similar previous reports by Poon et al.’s automatic rigid registration approach, which worsened the reconstruction (17% vs −48%). This is despite their experiment being conducted on a relatively levelled phantom structure to mosaic a fetus so small that it can be fully imaged in 6 volumes, and we have already established that heavily rotated US volumes acquired from complex shapes such as the shoulder are significantly more challenging to register than volumes acquired from levelled structures. Despite employing a similar interpolation method, our maximum compounding protocol also required significantly less time than average compounding (2 min vs 2s). Regarding limitations, their deformable registration approach significantly outperformed our hybrid registration in reconstruction accuracy improvements (17% vs 85%). It also showed no visible malalignments in the phantom. However, their approach remains unchallenged on a larger number of heavily rotated volumes [10]. Another limitation is that our approach required significantly longer registration time (33 min vs 32h). These findings show the comparative strengths and limitations of both mosaicking approaches.

This study’s **acquisition protocol** also introduces distinct strengths and trade-offs compared with **our previous** whole-shoulder mosaicking feasibility study based on expert manual registration [5]. The robot-stabilised, mechanically tracked setup improved sweep consistency and enabled broader anatomical coverage by distributing acquisition across additional regions, addressing limitations such as incomplete superior shoulder coverage and gaps previously reported near the ACJ [5]. However, despite closing prior gaps and being designed to ensure inter-regional overlap, some overlaps were not registration-informative because shared structures were acquired under substantially different viewing angles. This design also altered the overlap topology of the dataset: the robot’s kinematic constraints necessitated separating portions of the inferior shoulder into different sweeps, reducing the availability of direct inter-regional overlap and increasing reliance on boundary volumes to establish connectivity. While the longer protocol increased operational burden (45 min vs 25 min per trial), the hybrid refinement reduced total reconstruction effort relative to fully manual mosaicking (≈32 h vs > 240 h), improving practical scalability even though a direct accuracy comparison with [5] is not possible due to differences in evaluation strategy.

The **limitations** of this study largely reflect challenges in in-vivo US image registration as a whole rather than being study-specific. First, the absence of gold standards and ground truths in in-vivo US image registration hinders the evaluation and comparison of mosaicking and registration approaches. Studies rely on application-specific validation metrics; no universal method exists to assess registration accuracy. Several review papers have been published specifically to address the issue [34]. Second, the lack of reliable real-time 3D US imaging hardware technologies. Both 3D mechanical and phased array probes balance real-time imaging with image quality and detail, where real-time imaging or quick sweeping significantly diminishes resolution. This study prioritised image quality, using the long probe sweeping times (5s), which in turn prolonged experiment durations and increased involuntary patient movements. Future research may explore real-time imaging with optical tracking of both the probe and patient via skin-mounted markers to compare the effect of the diminished resolution from quick sweeping. Future work may also explore using multiple distributed, synchronised US transducers to simultaneously image various MSK landmarks of the shoulder, as suggested by [6] and [35]. No clinical US system allows the employment of various US probes of transducers, which may significantly reduce or completely dismiss patient movement and shoulder structural change-related errors. Finally, the acquisition of the MRI in a different shoulder pose poses a significant increase in the registration challenge. Future studies may investigate using an identical MRI pose as the target for US multimodal registration instead of guidance.

**Taken together**, our results define a concrete target for scalable whole-shoulder mosaicking, spanning overlap structure, refinement strategy, and compounding choice. The ideal dataset should provide inter-regional redundancy comparable to intra-regional overlap (≈3-volume mutual overlap in our acquisitions), but critically this overlap must be registration-informative, i.e., shared bony landmarks captured under comparable viewing angles rather than substantially different probe orientations. Where the objective is to curate the highest-quality reference mosaics for training and evaluation, leveraging available MRI/CT as an anatomical prior and benchmarking reference is justified; however, with overlap-rich acquisition, the same framework can progressively transition toward US-only global refinement. Achieving such informative inter-regional connectivity without extending scan duration (and increasing motion sensitivity) favours deliberate boundary “connector” sweeps with controlled probe orientation around stable landmarks and, longer term, parallelised multi-transducer acquisition to capture neighbouring regions concurrently [6,35]. On the reconstruction side, connector volumes should be treated as shared anchors across adjacent regions, with MRI retained as a soft global plausibility reference when pose mismatch is expected, and compounding selected to match overlap density (weighted mean when redundancy is sufficient; maximum when sparse), consistent with extended-FOV compounding observations [10]. Finally, while **non-rigid** refinement is desirable for in vivo mosaics, it should be introduced only after establishing a robust **automatic rigid** alignment backbone (e.g., data-driven rigid registration), so that non-rigid registration corrects residual local inconsistencies rather than compensating for unresolved global misalignment.

## 5. Conclusion

In conclusion, this study demonstrated the feasibility of overcoming the limited field-of-view of conventional US to achieve large-extent, whole-shoulder 3D US reconstruction in vivo using a novel hybrid mosaicking workflow. By integrating mechanically tracked pose estimation with semi-automatic monomodal and expert-guided multimodal refinement, the proposed approach consistently improved anatomical intra-regional alignment accuracy relative to pose estimation alone, with an average improvement of approximately 17%. Beyond feasibility, this work provides important insight into the behaviour of image-based refinement for large-extent shoulder reconstruction. Refinement was shown to be effective within regions with sufficient overlap, while global reconstruction quality remained strongly constrained by sparse inter-regional overlap and involuntary patient motion. Evaluation of post-processing strategies further revealed that compounding performance is scale-dependent, with maximum compounding remaining most suitable for global reconstructions under limited overlap conditions. Overall, this study establishes a practical reference framework for large-extent musculoskeletal US mosaicking and clarifies the structural acquisition and overlap requirements necessary to achieve robust global reconstruction, thereby informing future methodological and hardware developments and supporting the scalable generation of high-quality 3D US mosaics required for data-driven reconstruction approaches.

## Supporting information

S1 Fig US Calibration Protocol: The breast phantom was scanned by emphasising all 6 degrees of freedom through 60 acquisitions: vertical movements (in and out of the phantom), side tilt (through side-to-side rotations), front tilt (through front-to-back rotations), side movements (through side-to-side horizontal translations), front movements (through front-to-back horizontal translations) and rotations about a vertical axis. Five volumes were acquired per protocol movement, except for the vertical movement, which reduced three acquisitions, and the rotations increased to 6. The whole protocol was repeated twice.
(PNG)

S2 Fig Sonography Protocol: The protocol used by the sonographers during the experiments is shown in the anterior view (top) and posterior view (bottom) of the shoulder. Rounded rectangles and dotted arrow lines represent the US probe trajectory for every region, S1-S5 being the Anteroinferior, Anterosuperior, Posteroinferior, Posterosuperior, and Superior regions, respectively. Labels represent the anatomical structures used as the starting and ending points, and the dashed lines on bony anatomies represent the structures followed by the sonographer using the top or bottom of the probe during its travel.
(PNG)

S3 Fig Global visual results pose estimation. Reconstructed global ultrasound mosaics generated using calibrated robot pose estimation.
(PNG)

S4 Fig Global visual results hybrid. Reconstructed global ultrasound mosaics generated using the hybrid refinement workflow.
(PNG)

S1 Text Semi-automatic approach details. Detailed description of the semi-automatic registration workflow used during hybrid refinement [36,37].
(DOCX)

S2 Text Downsampling. Additional details describing voxel downsampling and volume compounding.
(DOCX)

S3 Text Segmentation details. Description of the humerus segmentation procedure.
(DOCX)
