# Supplementary figures and images for: Comprehensive reconstruction of the musculoskeletal anatomy in the shoulder using a hybrid 3D ultrasound mosaicking workflow: A pilot study
Source: PLoS One. 2026 Jun 9;21(6):e0347231. doi: 10.1371/journal.pone.0347231 (PMC13249142; doi:10.1371/journal.pone.0347231)

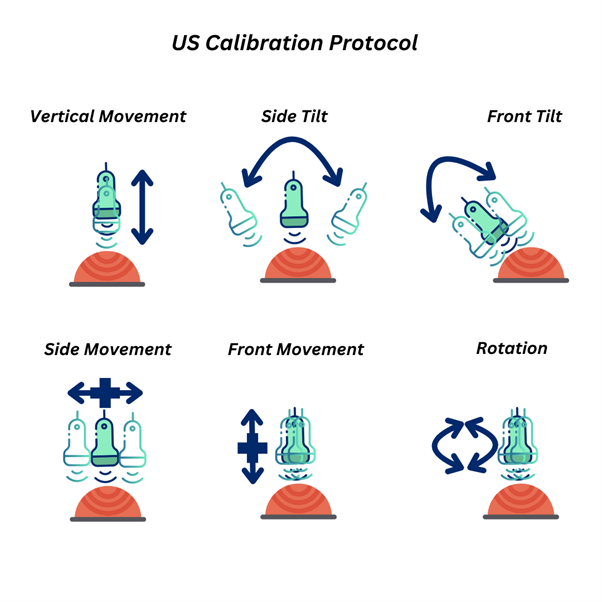

Supplement: S1 Fig — Five volumes were acquired per protocol movement, except for the vertical movement, which reduced three acquisitions, and the rotations increased to 6. The whole protocol was repeated twice. (PNG) [file pone.0347231.s001.png]

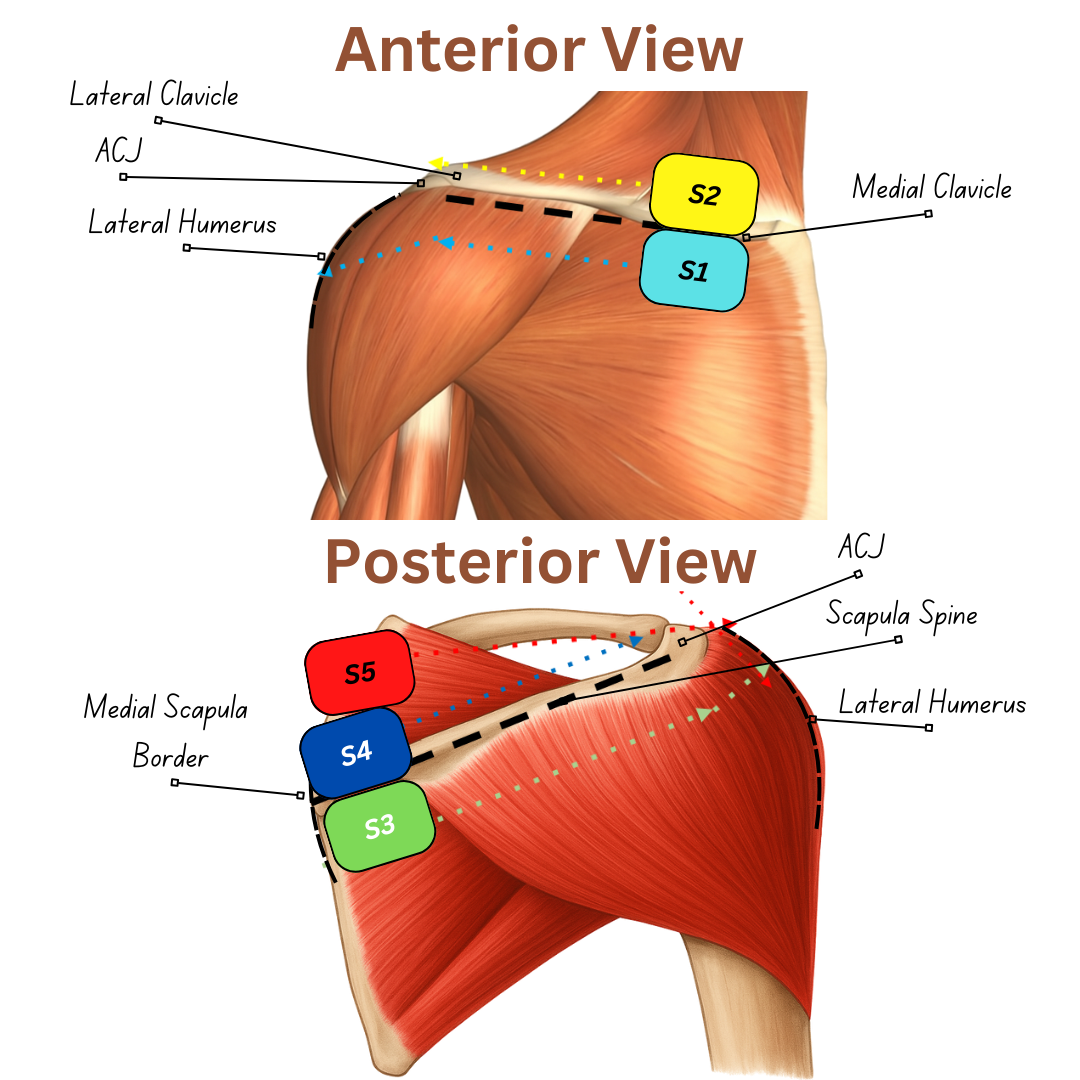

Supplement: S2 Fig — Rounded rectangles and dotted arrow lines represent the US probe trajectory for every region, S1-S5 being the Anteroinferior, Anterosuperior, Posteroinferior, Posterosuperior, and Superior regions, respectively. Labels represent the anatomical structures used as the starting and ending points, and the dashed lines on bony anatomies represent the structures followed by the sonographer using the top or bottom of the probe during its travel. (PNG) [file pone.0347231.s002.png]

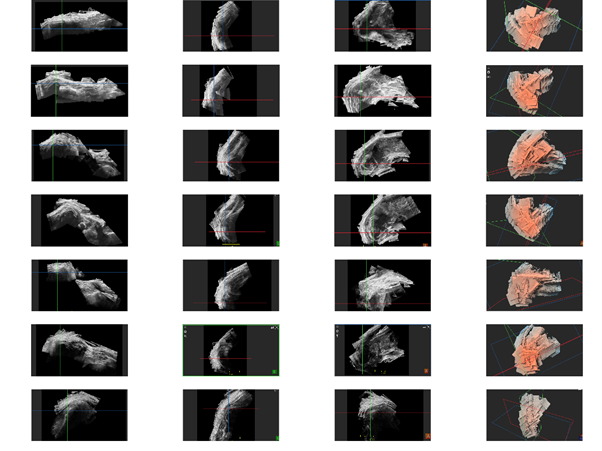

Supplement: S3 Fig — Reconstructed global ultrasound mosaics generated using calibrated robot pose estimation. (PNG) [file pone.0347231.s003.png]

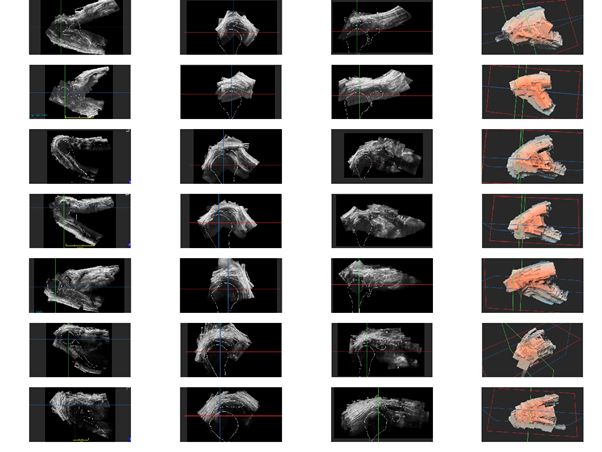

Supplement: S4 Fig — Reconstructed global ultrasound mosaics generated using the hybrid refinement workflow. (PNG) [file pone.0347231.s004.png]
