# Supplementary material for: Comprehensive reconstruction of the musculoskeletal anatomy in the shoulder using a hybrid 3D ultrasound mosaicking workflow: A pilot study
Source: PLoS One. 2026 Jun 9;21(6):e0347231. doi: 10.1371/journal.pone.0347231 (PMC13249142; doi:10.1371/journal.pone.0347231)
Supplement: S1 Text — Detailed description of the semi-automatic registration workflow used during hybrid refinement [36,37]. (DOCX) [file pone.0347231.s005.docx]

## S1 Text. Semi-Automatic Approach Details

The monomodal registration phase employed semi-automatic registration (referred to as SAMR), consisting of an automatic and an expert registration component. For the automatic registration algorithm, we opted for Fast NCC to overcome the computational cost of standard NCC [30]. The optimiser employed to minimise the parametric transformations according to the similarity measures was the Nelder mead simplex method [31] being a highly accurate, derivative-agnostic, simple algorithm with fast convergence. The algorithm and optimiser were applied using the ImFusion image registration toolbox. For the expert registration/intervention, the approach was similar to the one described in [5]. The operators aligned US volume pairs based on corresponding mutual MSK structures observed using their clinical knowledge, prioritising bony landmarks. The volumes were aligned based on the transverse, sagittal, and coronal planes and volumetric rendering display. The two techniques were cyclically performed until the operators deemed US volume pairwise alignment visually satisfactory. The expert multimodal registration involved the experienced operators manually aligning the US images according to the corresponding shoulder MRI also based on the corresponding structures observed in both modalities.
