# Supplementary material for: Comprehensive reconstruction of the musculoskeletal anatomy in the shoulder using a hybrid 3D ultrasound mosaicking workflow: A pilot study
Source: PLoS One. 2026 Jun 9;21(6):e0347231. doi: 10.1371/journal.pone.0347231 (PMC13249142; doi:10.1371/journal.pone.0347231)
Supplement: S2 Text — Additional details describing voxel downsampling and volume compounding. (DOCX) [file pone.0347231.s006.docx]

## S2 Text. Downsampling

Compounding large sets of high-resolution US volumes is a computationally expensive procedure that requires volume resampling and interpolation. Therefore, we downsampled target spacings between adjacent voxels, preserving the physical size of the volumes. The downsampling percentage was highly dependent on the number of volumes and the shoulder distance covered in each region. The IA and IP regions were the largest in both metrics, and the SS was the smallest. Hence, the volumes were downsampled as follows: 1–32 by 60%, 33–40 by 70% and 41–45 by 80%. The original size of each volume was 52MB.

After downsampling, all individual volumes were simultaneously compounded into a single US mosaic.
