# Supplementary material for: Comprehensive reconstruction of the musculoskeletal anatomy in the shoulder using a hybrid 3D ultrasound mosaicking workflow: A pilot study
Source: PLoS One. 2026 Jun 9;21(6):e0347231. doi: 10.1371/journal.pone.0347231 (PMC13249142; doi:10.1371/journal.pone.0347231)
Supplement: S3 Text — Description of the humerus segmentation procedure. (DOCX) [file pone.0347231.s007.docx]

## S3 Text. Segmentation Details

The segmentation was semi-automatically implemented in 3D Slicer (Texas, United States of America). Automatic thresholding was applied until the humerus was sufficiently captured. Manual adjustments were employed to paint the uncaptured humeral edges and erase unwanted segmentation islands. Unsegmented slices were automatically interpolated. Finally, smoothing was applied to fill residual holes and smooth the surfaces. Parameters of segmentation functions were consistently applied to all US volumes whenever applicable, and final segmentations were exported as STL files. Humeral segmentations were visualised under the same space using their respective volume poses, estimated from the two mosaicking methods.
